# Automated tracking to measure behavioural changes in pigs for health and welfare monitoring

**DOI:** 10.1038/s41598-017-17451-6

**Published:** 2017-12-14

**Authors:** Stephen G. Matthews, Amy L. Miller, Thomas PlÖtz, Ilias Kyriazakis

**Affiliations:** 1 Open Lab, School of Computing, Newcastle University, Newcastle upon Tyne, NE1 7RU UK; 2 Agriculture, School of Natural and Environmental Sciences, Newcastle University, Newcastle upon Tyne, NE1 7RU UK; 3 Present Address: Interdisciplinary Computing and Complex BioSystems (ICOS) Research Group, School of Computing, Newcastle University, Newcastle upon Tyne, NE1 7RU UK; 4 Present Address: School of Interactive Computing, Georgia Institute of Technology, Atlanta, GA 30318 USA

## Abstract

Since animals express their internal state through behaviour, changes in said behaviour may be used to detect early signs of problems, such as in animal health. Continuous observation of livestock by farm staff is impractical in a commercial setting to the degree required to detect behavioural changes relevant for early intervention. An automated monitoring system is developed; it automatically tracks pig movement with depth video cameras, and automatically measures standing, feeding, drinking, and locomotor activities from 3D trajectories. Predictions of standing, feeding, and drinking were validated, but not locomotor activities. An artificial, disruptive challenge; i.e., introduction of a novel object, is used to cause reproducible behavioural changes to enable development of a system to detect the changes automatically. Validation of the automated monitoring system with the controlled challenge study provides a reproducible framework for further development of robust early warning systems for pigs. The automated system is practical in commercial settings because it provides continuous monitoring of multiple behaviours, with metrics of behaviours that may be considered more intuitive and have diagnostic validity. The method has the potential to transform how livestock are monitored, directly impact their health and welfare, and address issues in livestock farming, such as antimicrobial use.

## Introduction

Monitoring behavioural changes in animals, including livestock, offers insights into the change of animal state. Such changes may be caused by health and welfare challenges, or changes in their environment^1^. Behaviour is an aid for identifying problems^2^ and changes can be revealed by direct observation of animals by their keepers during routine daily checks; for example, inability to stand, unresponsiveness to stimuli, or gross change in 24-hour feed consumption^3^. However, observation is impractical given the move towards large livestock settings that can house thousands of animals, to meet increasing global demand for food^4^. With substantially larger animal-to-staff ratios, the short periods of time for observation of each individual only permit detection of substantial changes in severely compromised animals, which may be too late for effective intervention. Observation of subtle changes that precede gross changes and clinical signs enables early detection^5^ and allows timely corrective action to be taken. For example, tail biting in pigs is a major compromise to their welfare, which affects production^6^, and increased restlessness can signal an outbreak up to six days in advance^7^, which on a commercial scale would be impossible to detect in the relatively short time for observations during daily checks.

Sensor-based automation methods to monitor animal behaviour longitudinally can overcome these challenges^1,8^. Monitoring with colour video cameras has identified changes in specific behaviours in groups of livestock by measuring optical flow patterns in commercial chicken houses^9,10^, or activity indexes in groups of pigs^11^ and chickens^12^. However, an animal may not respond to such challenges through change in a single behaviour^1^, so leveraging the full potential of video cameras to measure behaviours with known diagnostic validity is required for monitoring health and welfare challenges. 3D video cameras enable automated tracking and monitoring of animals capable of moving within their 3D environments, such as aquaculture^13^ and flying invertebrate^14^. Although pig movement occurs on the ground plane, 3D tracking enables measurement of standing behaviour, which is relevant to multiple health and welfare compromises. The practical use of depth sensors for automated monitoring is currently limited by manual calibration of starting positions for 3D tracking of pigs^15,16^ or thresholds for measuring standing from depth measurements^17,18^.

The objective was to develop an automated system with a single type of sensor–a depth video camera–to track 3D pig positions and measure multiple behaviours non-invasively that specifically have diagnostic validity. Standing behaviour is automatically modelled from the sensor’s depth measurements and a method is developed for measuring feeding for group-housed animals. A controlled challenge study introduced a novel object into pens to validate the automated system against ground truth data. The behavioural changes were representative of real health and welfare challenges in a commercial setting with large numbers of animals. Group-level changes, such as the standing and feeding durations, and spatial distribution; i.e., use of specific areas of the pen, were expected to be affected by this challenge in a predictable manner. Our long-term aim is the development of an automated system to monitor pig groups on commercial farms. This will lead to a new paradigm of raising livestock and transform the management of health and welfare challenges that arise.

## Results

### Behaviour Observations

Comparisons of manual observations of behaviour were performed between days in which a novel object (newspaper) was provided (treatment) and time-matched control days (control), and also between times of the same day within group. Data produced are mean scores of behaviour duration, per 5-minute interval, with control and treatment days alternating between pens on two consecutive days for the 4 pens (n = 4 per control and treatment).

Before provision of the novel object (11:00–12:00, all times denoted as hh:mm), there were no differences in pig behaviour between the control and treatment days, with similar durations of standing, feeding, drinking, and making non-nutritive visits (NNVs), which are defined as a pig entering the feeding area (see Materials and Methods; Fig. 7a) without consuming feed (Table 1).

**Table 1:** Ethogram for behavioural annotations in controlled study of novel object provision.

Behaviour	Description
Standing	Pig has only feet (and possibly snout) in contact with the pen floor
Feeding	Pig with head in food trough
Drinking	Pig consuming water from one of the nipple drinkers
NNVs	Pig enters the feeding area with 2 or more feet, and then leaves the feeding area having not consumed any food

**Figure 1:**
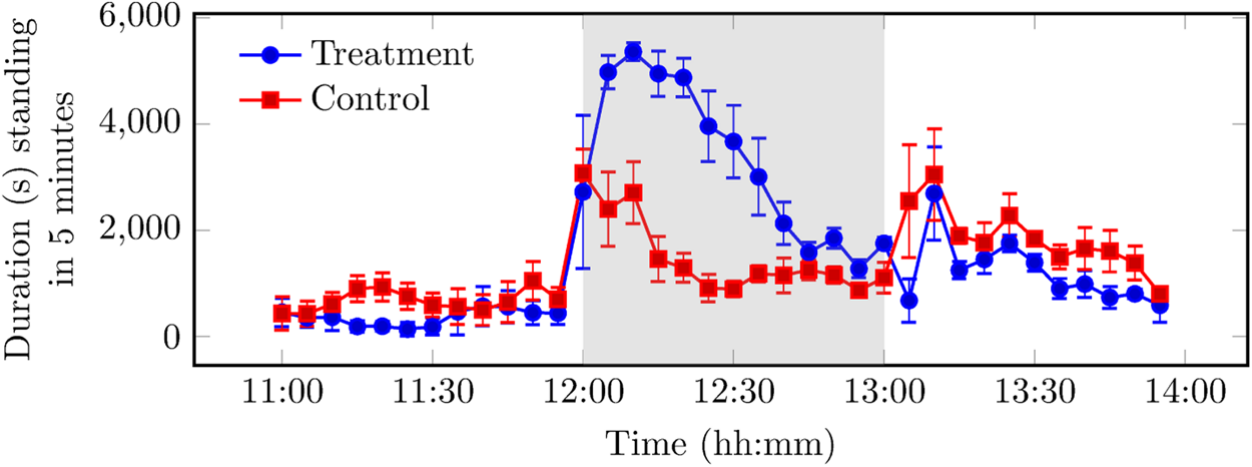
Observed standing duration (s, ± SEM) for control day, without intervention, and treatment day, where the novel object was introduced to the pen at 12:00. Each data point is the mean per 5-minute interval of 4 pens of pigs unless stated otherwise in Materials and Methods. Any remaining novel object was removed at 13:00. Data plotted represents pre (11:00–12:00), during (12:00–13:00, grey background), and post (13:00–14:00) provision of the novel object.

**Figure 2:**
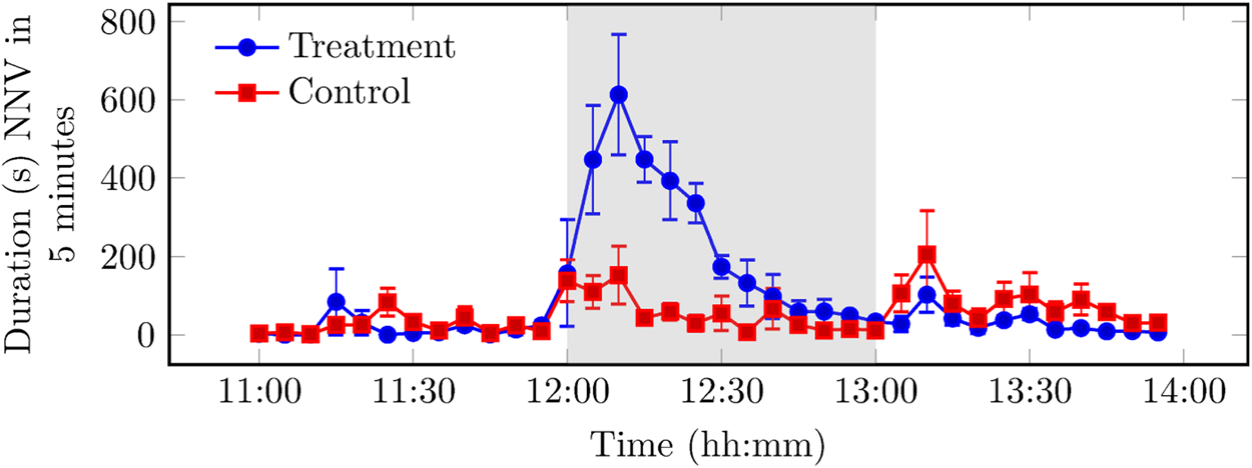
Observed non-nutritive visits (NNVs) duration (s, ± SEM) for control day, without intervention, and treatment day, where the novel object was introduced to the pen at 12:00. Each data point is the mean per 5-minute interval of 4 pens of pigs unless stated otherwise in Materials and Methods. An NNV was defined as a visit to the feeding area in the pen, where the pig then left the area having not consumed any food. Any remaining novel object was removed at 13:00. Data plotted represents: pre (11:00–12:00), during (12:00–13:00, grey background), and post (13:00–14:00) provision of the novel object.

**Figure 3:**
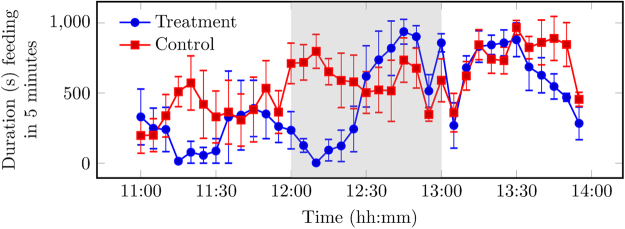
Observed feeding duration (s, ± SEM) for control day, without intervention, and treatment day, where the novel object was introduced to the pen at 12:00. Each data point is the mean per 5-minute interval of 4 pens of pigs unless stated otherwise in Materials and Methods. Any remaining novel object was removed at 13:00. Data plotted represents pre (11:00–12:00), during (12:00–13:00, grey background), and post (13:00–14:00) provision of the novel object.

During provision of the novel object (12:00–13:00), differences in the behaviour of the pigs on the control and treatment days were observed. On the treatment day, pigs became highly engaged with the novel object immediately following provision. During provision, standing duration was higher in comparison to the control day, as shown in the area under the curve (AUC) analysis of differences in behaviour (Fig. 1; AUC_treatment_ 255,020.40, AUC_control_ 80,416.30). On a control day, a human entered the pig building in the same manner as on a treatment day, but did not provide a novel object to the designated pens (See Materials and Methods). This caused the pigs to stand and then revert to prior baseline levels of standing within 20 minutes. Conversely, on the treatment day, the increased standing was sustained while the novel object was present. An increase in NNVs duration was observed during provision in comparison to the control day (Fig. 2; AUC_treatment_ 15,004.50, AUC_control_ 4,027.80). During provision, the feeding duration initially increased following a human entering the pig building on the control day. However, feeding duration remained similar to pre provision on the treatment day. The novel object was gradually destroyed by the pigs, and small amounts remained by 12:35. From 12:35 onwards, an increase in feeding duration was observed on the treatment day that returned to levels similar to the control day (Fig. 3; AUC_treatment_ 25,192.00, AUC_control_ 29,226.00). No differences were observed in drinking duration between control and treatment days (AUC_treatment_ 4,964.33, AUC_control_ 4,352.06).

Post provision of the novel object (13:00–14:00), an initial increase in the standing duration was observed in both control and treatment days in response to a human entering the pig building to remove any remaining novel object from the pens. Limited differences in behaviour were observed, with similar durations of standing, feeding, drinking and performing NNVs on both control and treatment days.

### Animal Tracking Performance

Data produced are means of all folds from 10-fold cross-validation (see Materials and Methods). The system tracked multiple pigs (19 pigs within the pen on the day of observation) to a precision of 0.06 (±3.0 ×10^−4^ SEM, n = 10) Multi-Object Tracking Precision (MOTP)^19^ (see Materials and Methods). The overall accuracy of the tracker’s ability to follow multiple pigs over time was 0.89 (±1.57 10^−3^ SEM, n = 10) mean Multi-Object Tracking Precision (MOTP)^19^ (see Materials and Methods), which measured 8.17% undetected pigs (false negatives), detected 2.1% non-existent pigs (false positives), and 0.33% errors arising from switching track identities between pigs when in proximity. The average length of time a pig was tracked for (track duration) was 21.88 s (±4.08 SEM, n = 10), which demonstrates the tracks are not fragmented into lots of small tracks.

### Automated Behaviour Measurement

**Figure 4:**
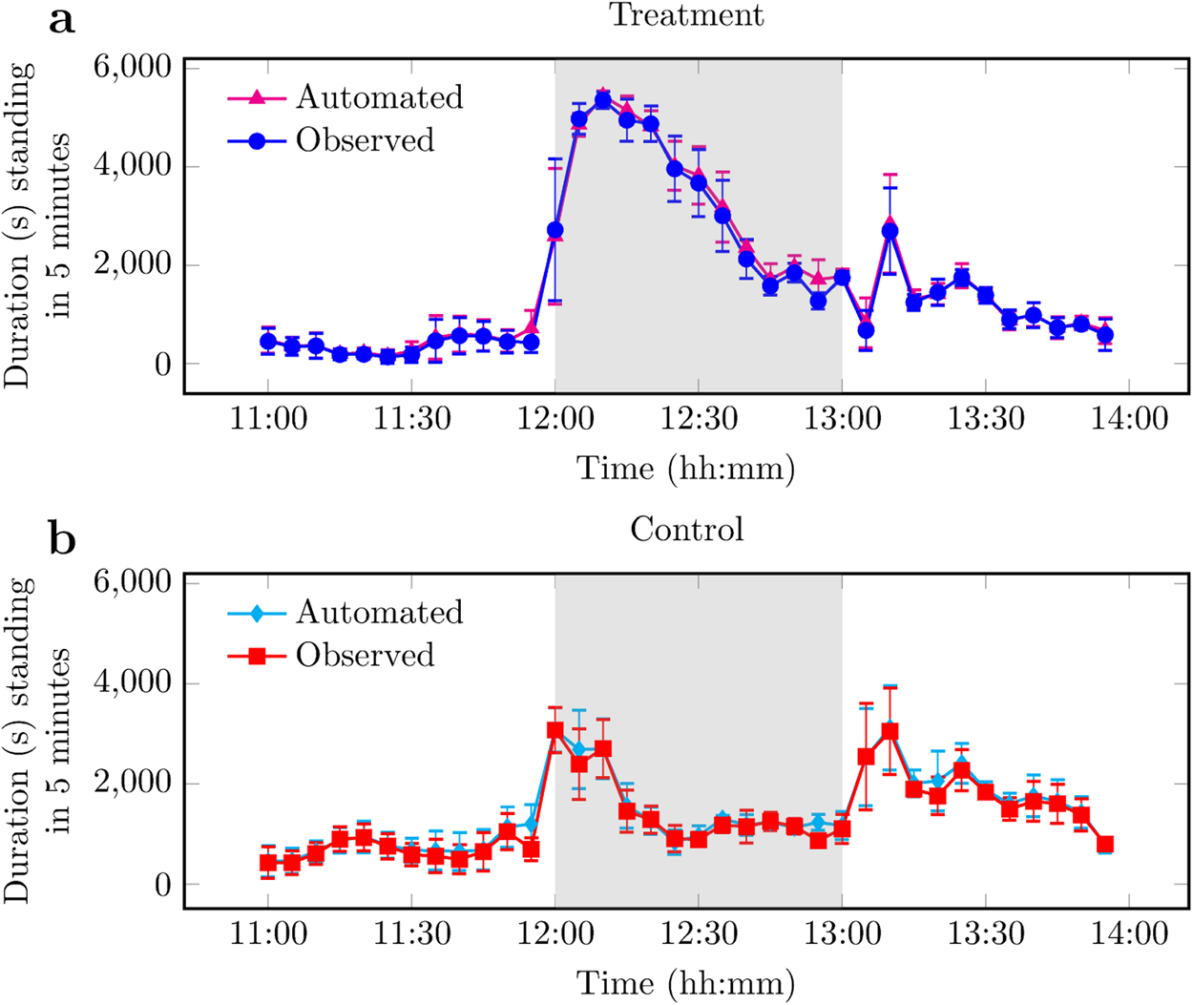
Automated and observed measurements of standing duration (s, ± SEM) for (**a**) treatment day, where the novel object was introduced to the pen at 12:00, and (**b**) control day, without intervention. Each data point is the mean per 5-minute interval of 4 pens of pigs unless stated otherwise in Materials and Methods. Any remaining novel object was removed at 13:00. Data plotted represents pre (11:00–12:00), during (12:00–13:00, grey background), and post (13:00–14:00) provision of the novel object.

A model of standing behaviour was developed from the sensor’s depth measurements of animal position. Gaussian Mixture Models (GMMs) were derived (see Materials and Methods) leading to two distinct model components that discriminated standing from not standing (e.g., lying and sitting; see Materials and Methods, Supplementary Figure S1, Supplementary Table S1). Models were derived for each day to account for change in animal size and for each pen to account for inter-group variation. Automated measurement of standing (Fig. 4) was positively correlated to ground truth data (number of pigs standing counted per frame) from the behaviour observations (Fig. 1) with ${R}^{2}$ (mean $\pm$ SEM, number of pens): control 0.94 $\pm$ 0.02, n = 4; treatment 0.97 $\pm$ 0.01, n = 4. The mean root-mean-square error (RMSE) was 128.71 s (n = 4, [1.93, 2483.68]) for control and 102.60 s (n = 4, [72.64, 2603.143]) for treatment.

The GMM for pen 4 on the treatment day (Supplementary Figure S1, top right figure) showed components with similar heights for standing and not standing. There was a small reduction in frames between 11:00–12:00 and 13:00–14:00 due to technical issues. The pigs were less active (longer duration of not standing) outside of the novel object provision between 11:00–12:00 and 13:00–14:00, and this is evident in the GMM with fewer $Z$ measurements around 1.85 m (lower red peak for not standing). The reduction in frames does not affect measurement of behaviours; for example, a pig still travels 1 m or feeds for 1 s across 5 depth frames if the middle frame is dropped.

**Figure 5:**
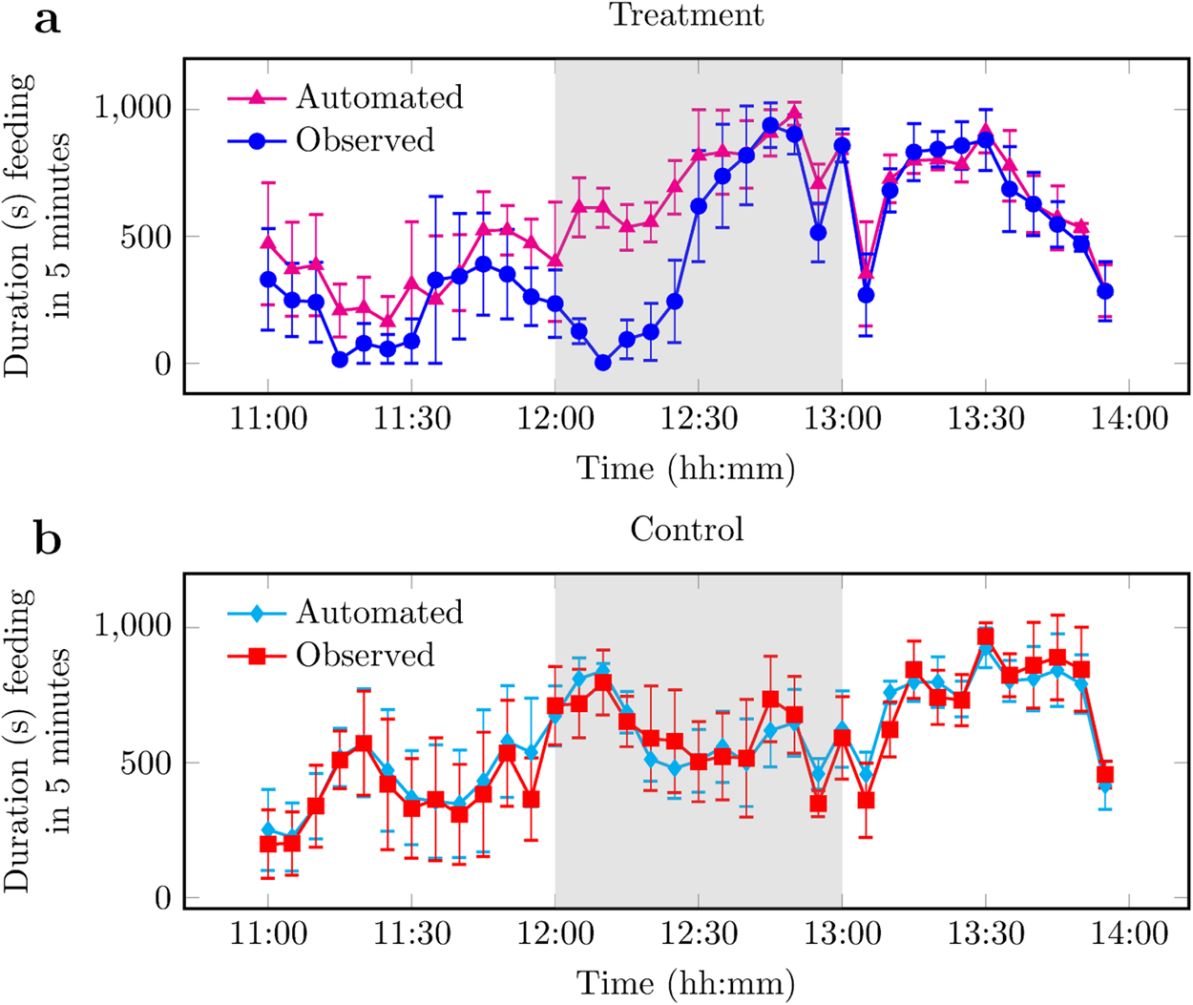
Automated and observed measurements of feeding duration (s, ± SEM) for (**a**) treatment day, where the novel object was introduced to the pen at 12:00, and (**b**) control day, without intervention. Each data point is the mean per 5-minute interval of 4 pens of pigs unless stated otherwise in Materials and Methods. Any remaining novel object was removed at 13:00. Data plotted represents pre (11:00–12:00), during (12:00–13:00, grey background), and post (13:00–14:00) provision of the novel object.

Automated measurement of feeding (Fig. 5) was positively correlated to ground truth data from the behaviour observations (Fig. 3) with ${R}^{2}$ (mean $\pm$ SEM, number of pens): control 0.86 ± 0.01, n = 4; treatment 0.49 ± 0.10, n = 4. The mean RMSE was 69.08 s (n = 4, [0, 576.61]) for control and 121.16 s (n = 4, [86.61, 576.21]) for treatment.

Automated measurement of drinking had no correlation with ground truth data from the behaviour observations with *R* ^2^ (mean ± SEM, number of pens): control -0.51 ± 0.28, n = 4; treatment -0.08 ± 0.06, n = 4. The mean RMSE was 44.92 s (n = 4, [0, 77.07]) for control and 38.76 s (n = 4, [0.47, 91.29]) for treatment.

**Figure 6:**
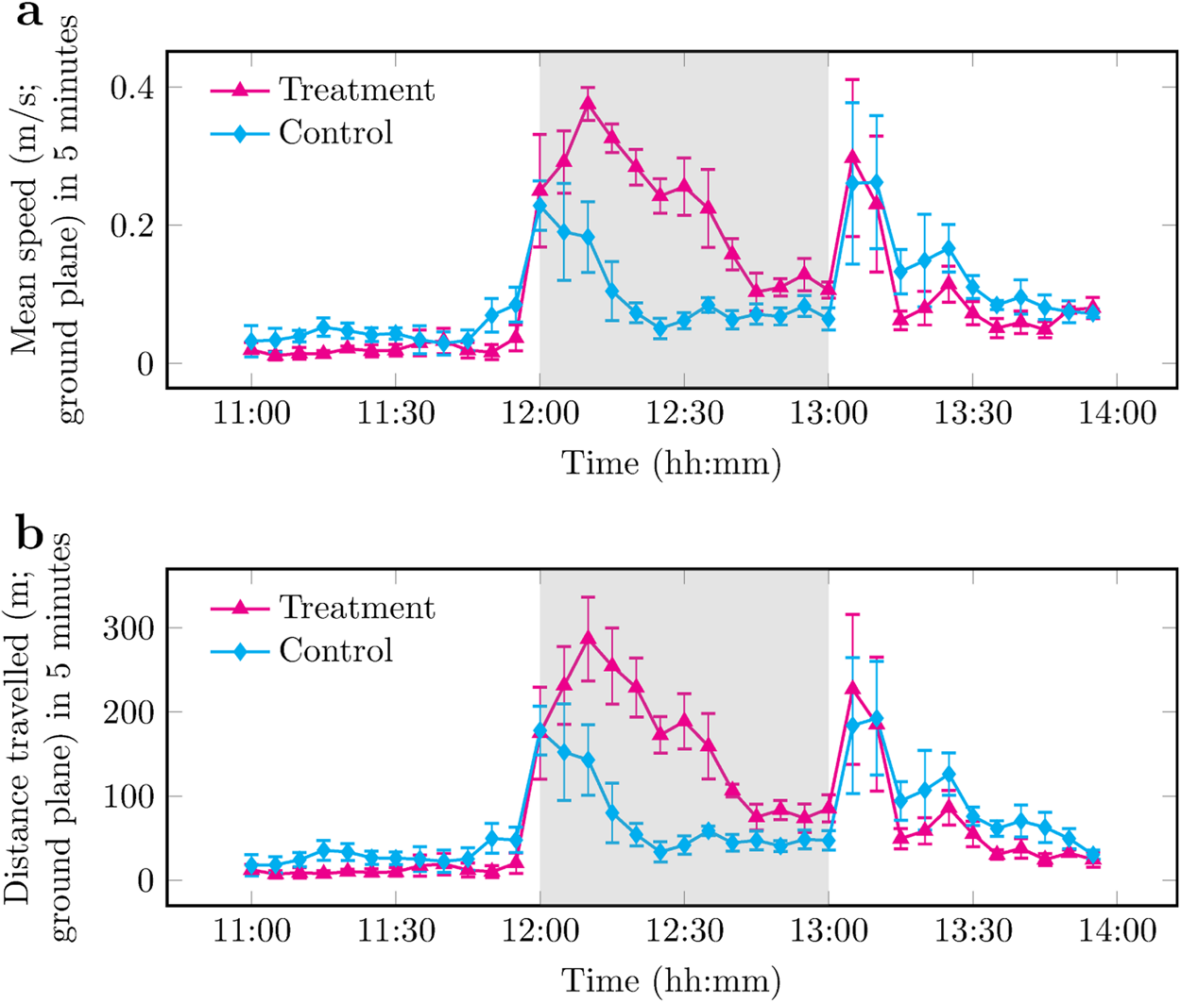
Automated measurement of locomotor activities for control day, without intervention, and treatment day, where the novel object was introduced to the pen at 12:00. Each data point is the mean per 5-minute interval of 4 pens of pigs unless stated otherwise in Materials and Methods. Any remaining novel object was removed at 13:00. Data plotted represents pre (11:00–12:00), during (12:00–13:00, grey background), and post (13:00–14:00) provision of the novel object. (**a**) Speed (m/s on ground plane, ± SEM). (**b**) Distance travelled (m on ground plane, ± SEM).

For locomotor activities, increased speed and distance was observed in both control and treatment days as a result of a human entering the pig building to provide the novel object to designated pens at 12:00. Locomotor activities on the control day subsided while activities on the treatment day were sustained by the novel object (Fig. 6). No differences were observed in spatial entropy between control and treatment days, which suggests pig use of space was unaffected by the novel object.

## Discussion

The need for continuous monitoring of animals in commercial settings is becoming increasingly important for mitigating risks in human food production, such as reductions in livestock performance and deterioration in their health and welfare^20^. Continuous monitoring of behaviour enables early identification of subtle changes that would otherwise be impossible for a human observer with intermittent monitoring on a commercial scale. The potential impact of early corrective action stands to improve the effectiveness of treatment, enhance animal welfare^21^, support profitable and sustainable production systems^22^, and may even have an impact on antimicrobial inputs^23^. A practical automated monitoring system was developed with a single type of sensor specifically for measuring multiple behaviours that change and have diagnostic validity. A controlled behaviour study validated a short-term change in group-level behaviour through the provision of a novel object, in a manner representative of a real-world scenario. Together, the automated system and controlled study provide a framework for developing understanding into changes in animal behaviour and early warning systems.

Evaluation of automated tracking against ground truth annotations demonstrated good performance in detecting individual animals and tracking over consecutive frames. The average length of time a pig was tracked for (track duration) was lower compared to a study that tracked group-housed pigs using a colour video camera evaluated on three pigs in a pen with more unoccupied space^24^. Detecting individual animals using 3D shape is beneficial for animals housed with commercial stocking density, which is a challenge of tracking multiple animals in proximity^25^. Tracking pigs with video provides more accurate position measurements than current RFID-based localisation methods^26^. The method for measuring standing from the depth camera is data-driven and does not require calibration, such as setting depth thresholds^17,18^. The method automatically learns models using depth data to label the measurements as standing or not, and improves upon quantifying the vertical activity from depth measurements by automatically labelling the data as standing^27^. For this study, standing is modelled for each day; however, in a production system, the frequency of retraining is expected to be less, and the models could be adapted rather than retrained.

Behavioural observations pre, during, and post provision of the novel object confirmed changes in durations of feeding, NNVs, and standing. Validation of the automated system’s ability to measure behaviour compared to manual observations demonstrated the suitability of these behaviours for monitoring changes. The method for measuring feeding is suitable for commercial settings because feeding is registered only when an animal is standing in front of the feeder, which overcomes limitations of pigs lying in this area without feeding^28^ and only using spatio-temporal clustering with fewer animals^29^. However, this method and RFID approaches^28^ do not filter pigs standing at the feeder and not feeding. In addition, the method may be more suitable in large-scale settings, where automated feeders that register the feeding behaviour of animals may be impractical or too expensive. Pigs spend a lot of time resting, so standing is an important measurement of activity. Automated measurement of animals with a different pattern of resting behaviour may not require measuring standing; for example, mice activity may be assessed from only speed of movement and turning angle^29^. Locomotor measurement is independent of animal growth because it uses the centroid of a pig, whereas methods based on the number of pixels include pig size in either activity measurement^11^ or spatial distribution^27^. Sensors attached to pigs, such as accelerometers, can measure standing behaviour^30^, but video cameras can be more practical.

The duration of feeding, standing and measurement of locomotor activities were relevant in this study because of the general diagnostic validity for multiple health and welfare challenges where behavioural intensity may increase or decrease. For example, infectious diseases associated with reduced feeding behaviour from pneumonia outbreaks^31^, reduced standing behaviour from Salmonella infections^32^, and reduced locomotion from parasitic infection^33^. NNVs demonstrated potential as a general diagnostic from the changes observed. NNVs are thought to be carried out by animals to facilitate or update their knowledge of food resources^34^. Reduced NNVs have been shown in calves with respiratory disease^35^ and mouse models of Alzheimer’s and Huntington’s disease^36–38^. In addition, identification of increased NNVs could also benefit monitoring; for example, in commercial settings, NNVs will increase with restlessness as observed here, and may be the case in tail-biting outbreaks. Automated measurement of NNVs is a limitation of the system, because the stay point method does not capture the context of what the pig is doing while staying in the area; i.e., it only detects a pig staying in that area and not that it hasn’t fed and left the area. A human provides accurate annotations of NNV from high resolution videos, but the automated method relies on lower resolution data from the pre-processed point clouds.

The protocol for the challenge study required humans to enter the pig building to retrieve any remains of the novel object after the one-hour period of its provision. These short, time periods were removed from the analysis because humans blocked the camera’s view of the pigs, which could be automatically removed in practice (e.g., excluding data from known observation routines), and should be considered when adding further automated analysis to the data. Also, changes in behaviour were expected as a result of a human entering the pig building to provide and retrieve remains of the novel object, which causes disruption in all pens (both control and treatment). This was observed as increased feeding duration on the control day, which was caused by the disturbance from resting, prompting the decision to feed after a period of rest. In the same manner, after a period of activity with the novel object on the treatment day, the pigs switched attention to feeding after the novel object was destroyed.

Whilst the system provides monitoring of behaviours to enable early corrective intervention, it also has some limitations. The low correlation of drinking behaviour measurements with observations may be caused by the nipple drinkers being very small and the high accuracy with which annotations were made. From an automated perspective, drinking was sometimes recorded when the pigs head was in close proximity to the drinkers, rather than specifically the mouth. Automated measurement of feeding does not distinguish visits that are feeding from visits that are not feeding. A non-feeding visit was defined as NNVs in the manual behavioural observations. Correlation of feeding behaviour measurements with observations were lower on the treatment day, which may be caused by increased activity in the feeding area when pigs are stationary and distracted from feeding by interacting with the novel object. Also, there were instances where the novel object was nudged into the feeder (for example, Supplementary Figure S2). More generally, the correlation of feeding may be lower compared to laboratory studies^29^, because higher stocking density in commercial settings causes pigs to stand next to the feeder when not feeding, which also occurs when measuring feeding with RFID in groups of pigs^28^.

In this study, behaviours are measured at the group level. However, measuring individuals is beneficial for confining detection of specific changes; e.g., one pig starts to feed less, and this is possible with RFID-based feeding systems^28^. There is a trade-off in sensor choice between the range of different behaviours that can be measured and measuring the behaviours of individuals or groups^1^.

The automated method’s practical nature in a commercial setting is significant for the following reasons. Automation is the key practical benefit that enables: continuous monitoring of large numbers of animals supported by human observation, objectivity by removing inter- and intra-observer variability, and identification of subtle behavioural changes that are not possible for a human to detect at the pen side. The method inferred multiple behaviour types from animal tracks (feeding, standing, and locomotor activities), and this multi-behaviour approach is more common for behavioural assays^13,15,39–42^. However, the tendency in livestock applications has been to focus on one specific behaviour at a time, such as social behaviour^15^, feeding and drinking^43^, aggression^18,44^, and locomotor activity^45^. Monitoring a single behaviour may be a risk for detection of health and welfare of groups, considering the variability from an individual animal’s response to a challenge and the environment. The system reports behaviours with metrics that may be considered more intuitive and unambiguous (i.e., time, distance, and speed) than those for current livestock monitoring based on abstract metrics, such as activity indexes and optical flow measures^9,11,46,47^.

The automated method supports the development of early warning systems. Recent progress focuses on monitoring behaviour where the responsibility of detecting problems falls upon users of the system^1^. Developments towards systems that automatically alert staff to behavioural changes are limited to threshold methods, which may be arbitrary, or focus on single behaviours^48,49^. Early warning systems should be robust to biological variation in individuals, the dynamics of different pens and groups, behavioural changes arising from natural growth, and ultimately false alarms that may otherwise require further analysis for alarm reduction and prioritisation^50^. To support these developments, behaviours are reported with diagnostic validity from a reproducible framework comprising a controlled behaviour study and automated behaviour monitoring system that is practical and relevant to commercial livestock. There is broader significance for the one-health concept where human, animal and environment health are all linked^51^.

## Materials and Methods

All procedures were conducted in accordance with the Animals (Scientific Procedures) Act 1986, European Directive EU 2010/63, and with the approval of the Newcastle University Animal Welfare and Ethical Review Body.

### Animals

**Figure 7:**
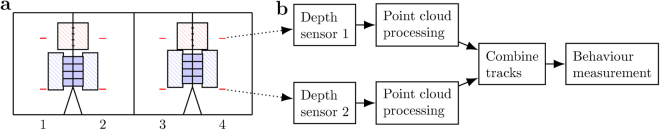
Automated tracking and behaviour system. (**a**) Pig building layout with depth sensors (red) set up in pens 1–4 in relation to feeders (light blue, 4 bins in each), feeding areas (Cross hatching not shown, but seems to be present in submitted eps), drinkers (4, short black horizontal lines above feeders), and drinking areas (Cross hatching not shown, but seems to be present in submitted eps). (**b**) Workflow illustrating major steps of tracking and behaviour measurement system.

Seventy-six pigs (Landrace/Large White × synthetic sire line, Hermitage Seaborough Ltd., North Tawton, UK) in mixed-sex groups that were approximately 9 weeks of age from the resident herd at Cockle Park Farm (Newcastle University) were used. All pigs were ear tagged at the start of the study for identification. They were randomly allocated in equal numbers to one of four identical fully slatted home pens (4.02 m long, 2.36 m wide, 2.14 m high) in a pig building (Portable Piggy Cabin, Jetwash Ltd.; Fig. 7a). Food and water were provided ad-libitum in each pen throughout, from 4 troughs and 4 nipple drinkers. A single plastic pipe suspended from a chain was provided to each pen as enrichment and remained present throughout. A seven-day acclimation period was given to the new pens prior to the start of the study. At the end of the study, all pigs returned to commercial stock at Cockle Park Farm (Newcastle University).

### Controlled Challenge

Behaviour of the pigs was temporarily disrupted through the provision of a novel object (a newspaper), in the home pen, for one hour (12:00–13:00). A human entered the pig building, where both control and treatment pens were present, and provided the novel object without entering the pen. The time chosen provided baseline data before the novel object was introduced, as pigs were less active during this time of day so a behaviour change would be more readily observable. Two randomly selected pens were provided with the novel object and the treatment was alternated between pens for two consecutive days for the 4 pens (i.e., n = 4 per group). The crossover design meant animals were their own time-matched controls.

### Equipment Setup

Two cameras (Microsoft Kinect for Xbox One) containing depth sensors were housed in ingress-protected enclosures and attached to the ceiling of each pen at a perpendicular angle to the floor (Fig. 7a). Depth sensors were positioned at 1.98 m from the ground to ensure all pigs were in view for automated tracking of position. The Kinect for Xbox One is a time-of-flight camera^52^ that uses infrared lasers to measure depth to an accuracy below 0.02 m up to distances of 3 m^53^, which improves upon the previous version of Kinect, also used for animal tracking^18^. The time-of-flight technology removes lighting conditions as a variable in sensing performance across the days in the controlled study. Each depth sensor was connected to one computer for data acquisition. Depth and infrared images were captured using the Kinect software development kit. Depth images were used for animal tracking. Videos for behaviour annotation were produced from the camera’s infrared stream and were encoded at 30 frames per second. Videos were split into 5-minute intervals. Data capture was synchronised across all cameras by time with Network Time Protocol.

### Behavioural Observations

Manual behavioural observations were carried out retrospectively using ELAN software (version 4.9.2, Max Planck Institute for Psycholinguistics, Nijmegen, The Netherlands) based on the ethogram in Table 1; only the behaviours that could be measured automatically were considered. The number of pigs performing each behaviour was recorded continuously during three key time phases were: pre (11:00–12:00), during (12:00–13:00), and post (13:00–14:00) novel object provision. Data were annotated for all pens on control and treatment days. Data were aggregated in 5-minute intervals and means of the 4 pens in each control and treatment day were produced.

### Analysis of Behavioural Observations

Data between control and treatment (novel object provision) days were compared. The duration of each behaviour (Table 1) was used for analysis. Area under the curve (AUC) analysis of behavioural change was conducted for the pre, during, and post provision time phases for the treatment day and time-matched control day. Data were plotted as total time of all pigs for each 5-minute interval against time of day. As 19 pigs were present in each pen, the total amount of time performing a given behaviour could exceed the real time value; e.g., two pigs standing for 5 minutes would quantify as a total time of 10 minutes.

### Quality Control

Data were not analysed for behavioural observations and automated behaviour monitoring between 13:05–13:10; i.e., when any remaining novel object was retrieved. Data were not analysed in pen 1 between 11:20–13:00 due to data loss.

### Animal Tracking

**Figure 8:**
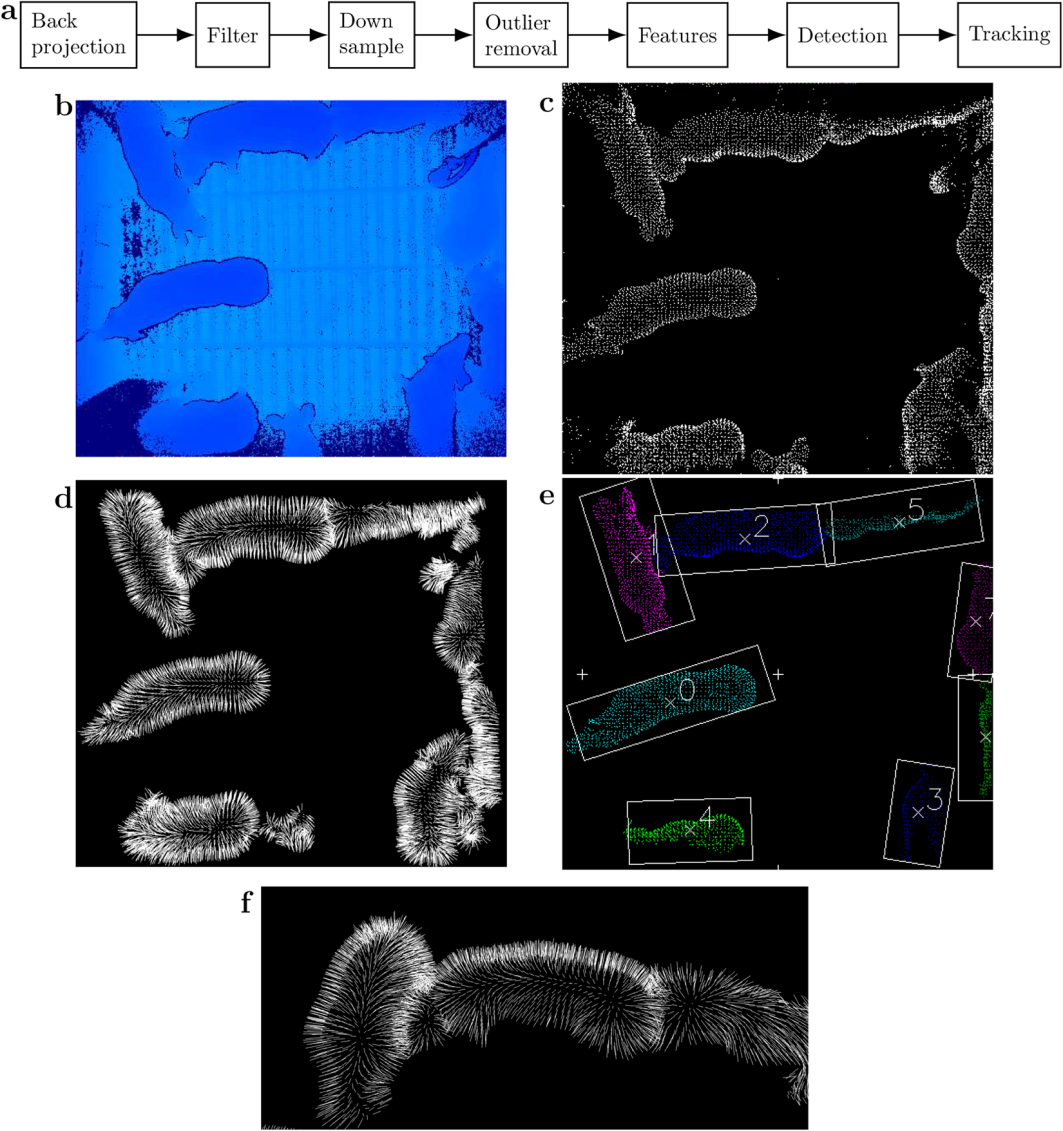
Workflow of 3D multi-animal tracking system with an example of processing frame. (**a**) Workflow illustrating detailed steps of post-acquisition processing of depth images in the multi-animal tracking system. (**b**) Example input depth image where the colour represents depth measurements. (**c**) Example of projected point cloud after back projection, filtering, down sampling, and outlier removal. (**d**) Example of surface normals in point cloud. (**e**) Example of detection and tracking. Detections are illustrated with oriented bounding boxes and × for centroids. Tracking is illustrated with numeric identifiers for each detection. (**f**) Example of discontinuity in surface normals with touching pigs.

Multi-animal tracking was performed by image processing on each depth frame (post-acquisition) from each camera to produce a series of time-stamped *XYZ* location points of all animals for behaviour measurement (Fig. 7b). The 3D multi-animal tracking system (Fig. 8) is summarised as follows.

The depth sensor produces images where each pixel is a distance measurement to objects in the environment. Depth images measured by the sensor were 2D (representative example in Fig. 8b) so they were converted (by back projection) to 3D point clouds (representative example in Fig. 8c). Each point in a point cloud is a single measurement of *XYZ* coordinates of the environment. Pre-processing of the point clouds and detection of pigs was performed with the Point Cloud Library (C++)^54^ and tracking of pigs over time was processed by custom C++ software.

Pre-processing of the point clouds involved multiple stages (Fig. 8a). The perpendicular angle of the camera, positioned on the ceiling, allowed filtering of the floor, walls, and pen gates by means of known *XYZ* coordinates. The result was a region of interest in the point cloud containing only pigs. The number of points in the cloud was reduced to allow reasonable processing time. For example, from the 5-minute validation dataset, the point clouds were down sampled from 217,088 maximal points to a mean of 10,476.71 points (±43.12 SEM). This was performed by dividing the cloud into a 3D grid and approximating the centroid of each 3D cell, known as voxels (0.02 m^3^). Measurement error from the depth sensor’s lasers were reduced by removing outliers. Outliers were removed if they fell outside a threshold, which was defined by the grand mean and standard deviation. The mean distance of each point local to its *k*-nearest neighbours (50) was used to calculate a grand mean from all the points. A standard deviation of 0.25 produced the largest mean track duration (Supplementary Figure S3a) with low MOTP (Supplementary Figure S3b) and high MOTA (Supplementary Figure S3c). Surface normals are a feature that was generated from the pig’s 3D shape. A surface normal is a vector that is perpendicular to every point on the pig’s surface (representative example in Fig. 8d). This feature emphasises the smooth shape across an animal’s body, but also the difference between touching animals based on the discontinuity of surface normals (representative example in Fig. 8f). Surface normals were approximated for each point on each pig. Points used in the approximation were inside a 0.06 m radius, which provided a level of detail suitable for representing pigs and the discontinuity between touching pigs.

Each animal was detected using region growing^55^. Region growing groups surface normals together that are similar. The surface normals from a single pig are grouped into the same region/detection because the pig’s smoothness leads to surface normals that are similar when next to each other. Pigs were tracked by linking detections of pigs from one frame to the same pigs in consecutive frames. The Hungarian algorithm^56^ performed this assignment between frames by performing a combinatorial optimisation of all pig-to-pig assignments. The optimal assignment has the least error in distance between centroids of pig detections (representative example in Fig. 8e).

Track coordinates were relative to each camera, so coordinates from cameras in the same pen were combined by their offset of ceiling positions (Fig. 7). Tracks were smoothed with a median filter with window size 5.

The frame rate for processing point clouds into tracks was set to 7 as this produced the largest mean track duration (Supplementary Figure S4a) with low MOTP (Supplementary Figure S4b) and high MOTA (Supplementary Figure S4c).

### Animal Tracking Validation

Performance was evaluated by comparing the tracker’s output with ground truth tracks annotated by a human observer. The images were annotated with rotated bounding boxes around every pig location and identities of pigs between images using custom HTML and JavaScript software. The images were point clouds projected along the camera’s *Z* coordinate. The projected images were coloured to emphasise contrast between nearest and furthest points in the *Z* dimension. A comparison of bounding box centroids from tracker hypotheses on the ground plane (*XY* camera coordinates) and corresponding ground truth annotations (after back projection from image to camera coordinates) quantified tracking system performance. Multi-Object Tracking Accuracy (MOTA)^19^ measures overall performance of the tracker. MOTA combines three sources of error and is calculated by equation (1):

1 $${\rm{MOTA}}=1-\frac{\sum _{t}({{\rm{FN}}}_{t}+{{\rm{FP}}}_{t}+{{\rm{IDSW}}}_{t})}{\sum _{t}{{\rm{GT}}}_{t}},$$

where *t* is the time index of the frame, FN is the number of false negatives, FP is the number of false positives (erroneous detections when no animal present), IDSW is the number of identity switches, and GT is the number of ground truth pigs. Multi-Object Tracking Precision (MOTP)^19^ measures the localisation precision between all true positive detections and corresponding ground truth annotations as calculated by equation (2):

2 $${\rm{MOTP}}=\frac{\sum _{i,t}{d}_{t}^{i}}{\sum _{t}{c}_{t}},$$

where *c* is the number of targets (pig detections) that correspond to ground truth annotations at time index of frame *t*, and *d* is the distance between target *i* and its corresponding ground truth annotation measured as the overlap between oriented bounding box. A 5-minute set of annotated images and automated tracks were produced at 7 frames per second. This image dataset was manually selected from observation to include a variety of activity levels and behavioural changes expected in the observation study, such as sitting, lying, feeding, drinking, and locomotor activities. Manual tuning with 10-fold cross-validation was performed with folds defined as 30-second subsets of data where frame ordering remained intact.

### Automated Behaviour Monitoring

Processing tracks to measure behaviours was performed with custom Python software (version 3.6). Standing behaviour was modelled with the depth sensor’s measurement of the distance to each animal. Univariate GMMs were derived for the *Z* camera coordinate of all animal centroids. Two model components were set to represent standing and other behaviour (lying and sitting). A uniform random sample of 10,000 *Z* measurements from each day and each pen (mean of 37.33% sampled points) was used as training data. Defining samples by size instead of percentage produced execution times that are suitable for operation in practice. Univariate GMMs were derived using standard Expectation Maximisation^57^ algorithm with 100 maximum iterations, full covariance, and k-means initialisation. Each model was tested on the remaining depth measurements for the respective pen and day using the two-sample Kolmogorov-Smirnov (K-S) goodness of fit test (*P* < 0.01).

Spatial and temporal information of animal position was used to determine presence at the feeding and drinking areas^29^. The definitions of these areas were determined from the behaviour observations by ensuring pig locations were inside the areas. Stay points are geographic regions where people stay over a certain time interval, which is measured with GPS trajectories^58^. It is applied here to livestock trajectories to identify pigs staying in the same location without moving outside of a radius for a specified time interval. The radius of the stay point area (0.05 m) and time threshold (1 s) was chosen from preliminary evaluation to accommodate movement of a pig whilst feeding/drinking and also the tracker’s precision in locating a pig (0.06 m MOTP). The method was applied to animals that were standing in the feeding and drinking areas (Fig. 7a) to prevent measuring pigs lying in these areas that are not feeding or drinking. Combining the spatial and temporal data with standing behaviour enabled measurement of presence at feeding and drinking areas where stocking density of animals is higher in commercial farming environments than laboratories^29^.

Locomotion behaviour was measured with speed and distance (Euclidean) on the ground plane (*XY* coordinates) to prevent measuring vertical movements from the *Z* dimension, which may be caused from transitions to and from standing. Speed was measured from the cumulative between-frame distances traversed across 7 frames.

Spatial entropy *H* measured the distribution of the animal positions across the pen and is calculated by equation (3):

3 $$H=-\sum _{i}{p}_{i}\,\mathrm{log}\,\frac{{p}_{i}}{{q}_{i}},$$

where *p* is the number of animals in one two-dimensional bin defined by spatial interval *q* of the entire pen area^59^. The pen floor was binned into 3 horizontal and 6 vertical spatial intervals.

### Automated Behaviour Monitoring Validation

Data between behavioural observations and automated behavioural measurements were compared. The duration of each automated behaviour was used for analysis. The coefficient of determination *R* ^2^ between both was performed and reported with standard error of the mean to assess correlation. The goodness of fit was tested with an *F*-test (*P* < 0.001) and a null hypothesis that the fit of the behaviour measurement model without independent variable is equal to the measurement model. Root-mean-square error (RMSE) was performed and reported with range to assess accuracy. The analysis was performed using Python (version 3.6) with the scikit-learn (version 0.19.0) and SciPy (version 0.14.0) libraries. Data were plotted as total time of all pigs for each 5-minute interval against time of day.

## Electronic supplementary material

Supplementary Information
