# Supplementary material for: Automated tracking to measure behavioural changes in pigs for health and welfare monitoring
Source: Sci Rep. 2017 Dec 14;7:17582. doi: 10.1038/s41598-017-17451-6 (PMC5730557; doi:10.1038/s41598-017-17451-6)
Supplement: Supplementary file 1 — Supplementary Information [file 41598_2017_17451_MOESM1_ESM.pdf]

Automated tracking to measure behavioural changes in pigs for health and welfare monitoring

Stephen G. Matthews[1,3,+,*], Amy L. Miller[2,+], Thomas Plötz[1,4], and Ilias Kyriazakis[2]

[1]Open Lab, School of Computing, Newcastle University, Newcastle upon Tyne, NE1 7RU, UK
[2]Agriculture, School of Natural and Environmental Sciences, Newcastle University, Newcastle upon Tyne, NE1 7RU, UK
[*]stephen.matthews@newcastle.ac.uk
[+]these authors contributed equally to this work
[3]Current address: Interdisciplinary Computing and Complex BioSystems (ICOS) Research Group, School of Computing, Newcastle University, Newcastle upon Tyne, NE1 7RU, UK
[4]Current address: School of Interactive Computing, Georgia Institute of Technology, Atlanta, GA 30318, USA

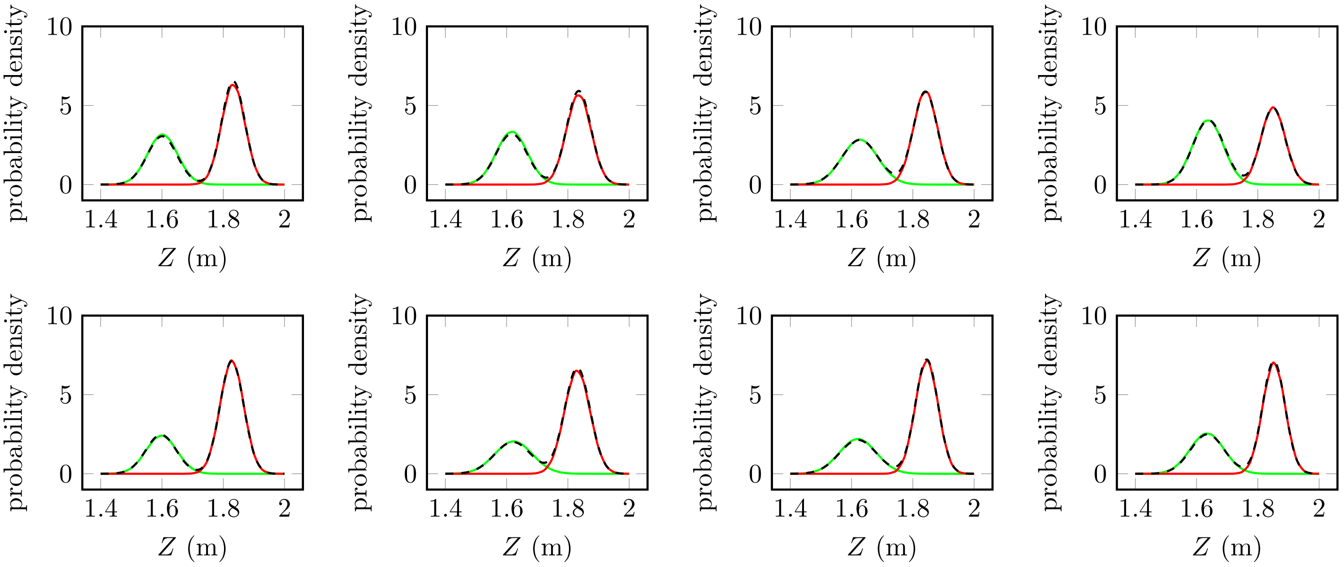

Supplementary Figure S1: Gaussian Mixture Models (GMMs) for measuring standing behaviour (green) and other behaviour (red; lying and sitting). Each GMM (dashed line) represents sensor depth measurements (Z) with components (solid lines) for standing (green) and not standing (red; sitting and lying). Depth measurements for all detected pigs per pen (left to right) and days (treatment top and control bottom.

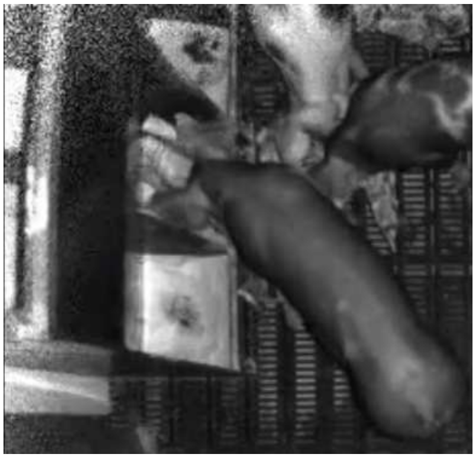

Supplementary Figure S2: Representative example of pig interacting with novel object in feeding bin. Image taken at 11:00:45 on treatment day from a pen that had low correlation between automated and observed feeding.

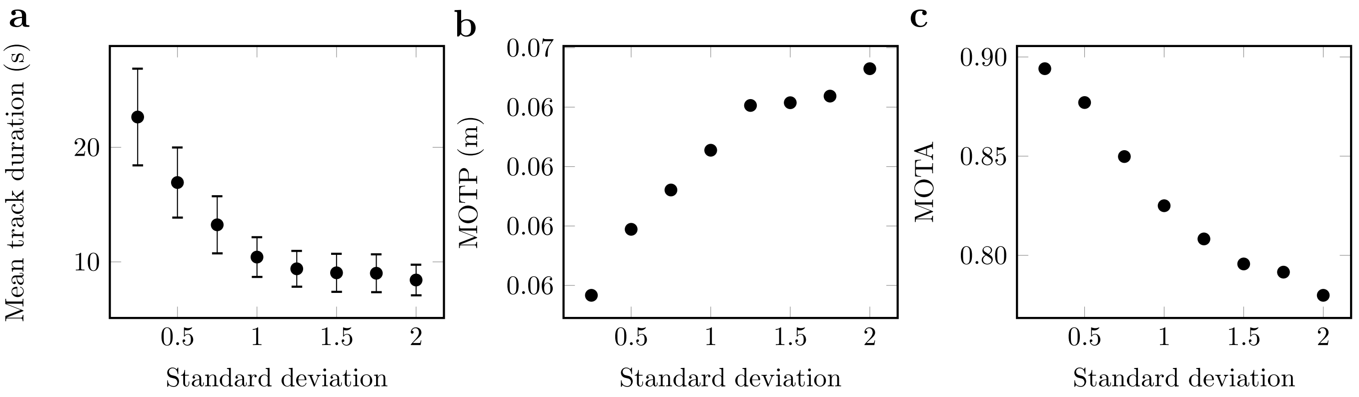

Supplementary Figure S3: Effect of standard deviation (of a point neighbourhood) in statistical outlier removal on tracker performance. Data from the 5-minute set of annotated images used in animal tracking validation. (a) Mean track duration (s, ± SEM) of all tracks in 5-minute set of images. (b) Multi-Object Tracking Precision (MOTP) metric measures distance between bounding box centroids of all true positive detections and corresponding ground truth annotations. (c) Multi-Object Tracking Accuracy (MOTA) metric aggregates false negatives, false positives, and identity switches.

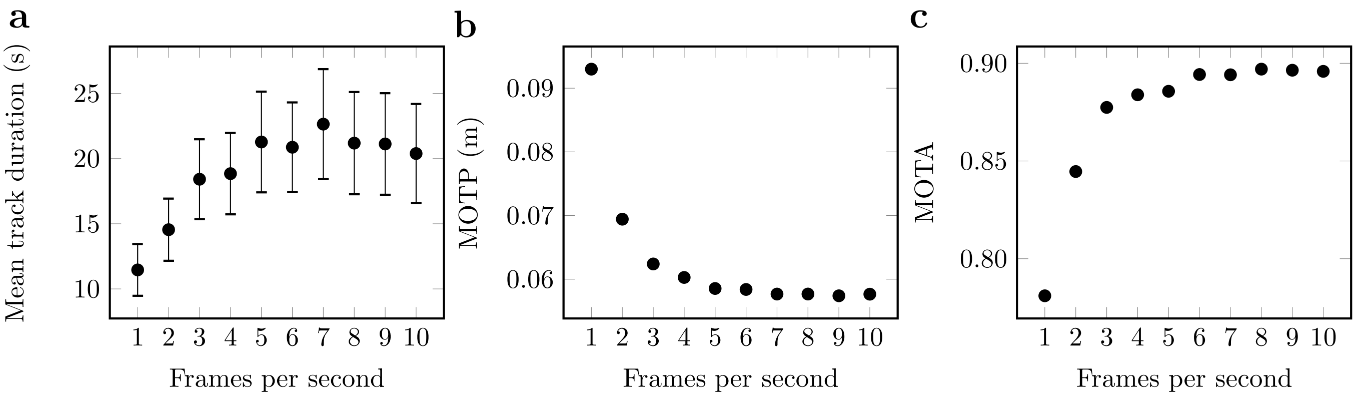

Supplementary Figure S4: Effect of frames per second on tracker performance. Data from the 5-minute set of annotated images used in animal tracking validation. (a) Mean track duration (s, ± SEM) of all tracks in 5-minute set of images. (b) Multi-Object Tracking Precision (MOTP) metric measures distance between bounding box centroids of all true positive detections and corresponding ground truth annotations. (c) Multi-Object Tracking Accuracy (MOTA) metric aggregates false negatives, false positives, and identity switches.

Day	Pen	GMM parameters
Treatment	1	$\mu = 1.60$, $\sigma = 0.049$, $\mu = 1.83$, $\sigma = 0.038$
Treatment	2	$\mu = 1.62$, $\sigma = 0.051$, $\mu = 1.83$, $\sigma = 0.040$
Treatment	3	$\mu = 1.62$, $\sigma = 0.059$, $\mu = 1.84$, $\sigma = 0.039$
Treatment	4	$\mu = 1.63$, $\sigma = 0.050$, $\mu = 1.85$, $\sigma = 0.040$
Control	1	$\mu = 1.60$, $\sigma = 0.050$, $\mu = 1.83$, $\sigma = 0.039$
Control	2	$\mu = 1.62$, $\sigma = 0.061$, $\mu = 1.83$, $\sigma = 0.042$
Control	3	$\mu = 1.62$, $\sigma = 0.061$, $\mu = 1.85$, $\sigma = 0.037$
Control	4	$\mu = 1.63$, $\sigma = 0.055$, $\mu = 1.85$, $\sigma = 0.037$

Supplementary Table S1: Gaussian Mixture Models (GMMs) of camera depth measurements (Z) of all detected pigs. Each model trained on 10,000 points and tested on remaining points. Parameters are for GMM components for standing and other (lying and sitting).
